# Vascular Resection for Intrahepatic Cholangiocarcinoma: Current Considerations

**DOI:** 10.3390/jcm10173829

**Published:** 2021-08-26

**Authors:** Ruslan Alikhanov, Anna Dudareva, Miguel Ángel Trigo, Alejandro Serrablo

**Affiliations:** 1 Department of Liver and Pancreatic Surgery, Department of Transplantation, Moscow Clinical Scientific Centre, 111123 Moscow, Russia; r.alikhanov@mknc.ru; 2 Department of Vascular Surgery, Moscow Clinical Scientific Centre, 111123 Moscow, Russia; a.dudareva@mknc.ru; 3 Department of Pathology, Miguel Servet University Hospital, 50009 Zaragoza, Spain; matrigo@salud.aragon.es; 4 HPB Surgical Division, Miguel Servet University Hospital, 50009 Zaragoza, Spain

**Keywords:** cholangiocarcinoma, liver resection, intrahepatic cholangiocarcinoma, survival, vascular resection

## Abstract

Intrahepatic cholangiocarcinoma (iCCA) accounts for approximately 10% of all primary liver cancers. Surgery is the only potentially curative treatment, even in cases of macrovascular invasion. Since resection offers the only curative chance, even extended liver resection combined with complex vascular or biliary reconstruction of the surrounding organs seems justified to achieve complete tumour removal. In selected cases, the major vascular resection is the only change to try getting the cure. The best results are achieved by the referral centre with a wide experience in complex liver surgery, such as ALPPS procedure, IVC resection, and ante-situ and ex-situ resections. However, despite aggressive surgery, tumour recurrence occurs frequently and long-term oncological results are very poor. This suggests that significant progress in prognosis cannot be expected by surgery alone. Instead, multimodal treatment including neoadjuvant chemotherapy, radiotherapy, and subsequent adjuvant treatment for iCCA seem to be necessary to improve results.

## 1. Introduction

Intrahepatic cholangiocarcinoma (iCCA) is a malignant tumour that develops from the epithelium of the bile ducts proximal to the second division. It is the most common primary biliary malignancy next to perihilar cholangiocarcinoma, with a trend of increasing incidence and mortality rates in Western countries in the last two decades [1,2,3]. Primary liver cancer mortality has become more sustained across Europe over recent years, with an overall decline; in contrast, iCCA mortality has substantially increased in most European countries, accounting for over a quarter of all liver cancer deaths in men and ~50% in women [4].

Among all detected iCCA cases, 60% of patients are not candidates for curative resection due to the presence of metastatic disease. At the initial evaluation, approximately 50% of patients are diagnosed with locally advanced invasion of the surrounding organs, including major vascular structures, such as the inferior vena cava (IVC) or portal vein (PV) [5,6]. A recent multi-centre study demonstrated that increased tumour number, vascular invasion, and lymph node metastasis were associated with decreased survival. A univariate analysis showed that the presence of vascular invasion was associated with worse survival; patients without vascular invasion had a median survival of 41.0 months versus 20.0 months for those with vascular invasion. N1 status adversely affects overall survival (OS) and influences the relative effect of tumour number and vascular invasion on prognosis [5,6].

The first clinical historical studies have demonstrated better survival with hepatic resection than conservative therapy if margin-negative resection (R0) can be achieved [7,8]. Without treatment, the median survival does not exceed 9 months, with a 5-year survival rate of 3% [9,10,11]. In this regard, interest has grown in the possibility of surgical treatment for cholangiocarcinoma with spread to the vascular structures. The biological significance of macro- and microvascular invasion (MVI), the difficulty with diagnosis and prognosis, and the possibility of performing radical resection and vascular reconstruction to increase resectability are relevant issues that require further assessment.

## 2. Pathology

Cholangiocarcinoma is currently classified both pathomorphologically and clinically for evaluation of prognosis and treatment strategy. According to the pathology determination, iCCA is most commonly an adenocarcinoma with biliary differentiation arising in any segment of the intrahepatic biliary tree from the peripheral periportal ductules and small portal bile ducts to the perihilar segmental ducts [12]. Based on its macroscopic appearance, the Liver Cancer Study Group of Japan [12,13,14] subdivided iCCA into four types: (I) mass-forming tumour, consisting of a greyish-white, well-delimited, firm, solid, non-encapsulated, and polylobulated mass within the liver at a distance from the hilum with no connection macroscopically discernible with a bile duct; (II) periductal infiltrating tumour, characterised by spreading along the intrahepatic portal tract, together with stenosis of the involved ducts and upstream bile duct obstructive dilatation and cholangitis; (III) intraductal growth tumour, typified by a polypoid or papillary tumour mass growing within the lumen of a dilated large bile duct; and (IV) mixed [15].

Imai et al. demonstrated different biological and clinical tumour behaviors depending on tumour type. The survival rate after surgery was significantly lower in the mass-forming type than in the periductal infiltrating type, with 5-year survival rates of 41.2% and 85.7%, respectively (*p* = 0.032). The periductal infiltrating type was most often associated with jaundice, intrahepatic biliary fibrosis, and cholangitis. PV invasion, lymph node involvement, and positive surgical margins occurred more frequently in the mixed mass-forming and periductal infiltrating types of iCCA [16].

Vascular invasion is a significant prognostic risk foractor for poor post-resection outcome in iCCA because it may indicate a higher tendency towards intrahepatic and extrahepatic metastasis [17].

Nakajima et al. [18] assessed the clinicopathological characteristics of 102 patients with iCCA. With regard to intrahepatic tumour spreading, expansion via the sinusoidal spaces (93%), vascular (52%) or lymphatic (18%) involvement, perineural invasion (16%), replacement of growth in the bile duct (12%), and permeation in the portal connective tissue (19%) were observed. Cholangiocarcinomas with vascular involvement presented a higher tendency toward intrahepatic and extrahepatic metastasis.

The role of the vascular involvement of iCCA and its correlation with other tumour characteristics, such as size and number of nodules, remains uncertain. The association between tumour size, microscopic and macroscopic invasion, and survival in patients with iCCA were evaluated in a multi-centre study of 443 patients who underwent surgical resection. Using univariate and multivariate analyses, the authors revealed that, with increasing tumour size, the incidence of microscopic vascular invasion also increased: <3 cm, 3.6%; 3–5 cm, 24.7%; 5–7 cm, 38.3%; 7–15 cm, 32.9%, ≥15 cm, and 55.6% (*p* < 0.001). Tumour size was also associated with worsening tumour grade. The presence of perineural invasion and regional lymph node metastasis was independently associated with an increased risk of microscopic vascular invasion in tumours ≥5 cm. The risk of microscopic vascular invasion and worse tumour grade increased with tumour size [17]. In our series, 40% of cases with radiologically suspected macrovascular invasion were not confirmed in the pathological analysis (Table 1, although it was impossible to determine the absence of involvement intraoperatively, and it could have been difficult to achieve R0 resection without vascular resection.

Further studies are needed to determine the role of vascular invasion and to predict the outcome of surgical treatment.

## 3. Diagnosis

The preoperative diagnosis of iCCA and its invasion into the vessels is extremely important because it determines staging, as well as the need to plan vascular resections and the risk of post-resection hepatic failure.

Currently available diagnostic methods for diagnosing iCCA and vascular involvement include ultrasound, contrast-enhanced computed tomography (CT), magnetic resonance imaging (MRI), and positron emission tomography CT.

Contrast-enhanced CT or MRI are the main diagnostic tools for determining the extents of locoregional disease, vascular invasion, and metastatic spread to assess resectability. Both CT and MRI scans have good discriminatory ability for assessing portal and arterial invasion, with magnetic resonance cholangiopancreatography properly defining biliary anatomy and tumour involvement [19,20]. Mass-forming iCCA usually has a diameter of 5–10 cm at the time of diagnosis, while intraductal iCCA is a slowly growing papillary tumour that is usually confined to the bile duct wall [21,22,23].

Vascular involvement is depicted in approximately 50% of cases and usually presents as an obvious enhancing tumour thrombus within the PV and/or hepatic veins (as vascular encasement or distortion) and more often concerns the portal branch than the hepatic veins. The presence of segmental or lobar atrophy is strongly associated with ipsilateral PV encasement. The accuracies of CT and MRI are high, and false-negative cases correspond to encasement of the segmental portal branches. Although these two examinations are comparable, vascular involvement is considered more visible on CT [24].

The preoperative MRI evaluation of a number of patterns may also indicate MVI. Among MRI characteristics, tumour morphology, intrahepatic duct dilatation, arterial phase enhancement pattern, visible hepatic artery penetration sign, maximum tumour diameter, and arterial phase edge enhancement ratio were correlated with MVI (*p* = 0.007, 0.003, 0.008, 0.000, 0.003, and 0.002, respectively). Furthermore, higher CA19–9 levels (≥37 U/mL) and pathological tumour grade III were also associated with MVI (*p* = 0.014 and 0.004, respectively) [25].

## 4. Surgery

Significant improvements in surgical techniques and intensive care have led to aggressive surgical management of iCCA with involvement of the PV or IVC [26]. Recent studies showed the feasibility of hepatectomy with vascular resection in patients with iCCA, demonstrating encouraging postoperative results [27,28].

An important aspect of the radical treatment of iCCA is the achievement of a microscopically (R0) negative margin. Li et al. reported that incomplete resection (R1/R2) is associated with a higher risk of recurrence and worse OS [29]. Vascular resection may increase resectability, thereby allowing negative tumour margins. Regarding the use of techniques to increase the volume of the future remnant liver, PV embolization, biembolization (PV embolization + hepatic vein embolization), and Associating Liver Partition and Portal vein ligation for Staged hepatectomy (ALPPS) have been used. In 2019 and 2020, one single-centre study and one multi-centre study analysed ALPPS results. ALPPS was not applied in either study when macrovascular involvement was suspected [30,31]. In contrast, in our unpublished series with biembolization series, it was used in two cases with R0 resection of the IVC and all hepatic veins.

The technical features of concomitant hepatic and vascular resection are important to achieve satisfactory long-term oncological results. The principles and techniques of PV resection in iCCA are similar to those in perihilar cholangiocarcinoma, including resection of the bifurcation of the PV and reconstruction by direct vascular anastomosis or, rarely, the use of different types of allografts [28]. Macrovascular tumour invasion adds technical complexity to surgical extirpation, but it can be achieved with en bloc vascular resection.

IVC procedure type depends on tumour location and caval involvement extent [32]. When IVC involvement is less than 60° circumferentially and ≤2 cm longitudinally, control may be achieved by the application of a clamp to the wall of the retrohepatic IVC to enable direct repair. To prevent IVC narrowing, a patch of bovine pericardium [32], peritoneum [33], or left renal vein can be applied [34]. Larger involvement of the IVC requires resection and replacement with a synthetic graft under total vascular exclusion. For these techniques, a deleted Dacron tube graft (Hemashield^®^; Meadox Medical, Inc., Oakland, NJ, USA) or ring-enforced polytetrafluoroethylene (PTFE) tube graft (Gore-Tex^®^; W. L. Gore & Associates Inc., Flagstaff, AZ, USA) may be used [35]. Although replacement of the resected IVC with an autogenous vein graft has advantages regarding the risk of infection or thrombosis, this technique may not be feasible in terms of a relatively long segment of the IVC being replaced; in such cases, synthetic grafts are preferable [32]. Dacron^®^ was widely used in the past but has been associated with relatively high thrombosis and stenosis rates, and the use of reinforced PTFE grafts for replacing the IVC has recently been recommended [32,35].

With complete vascular isolation, extracorporeal hepatic resection (ante- and ex-situ resection) can be used to reduce the risk of hemodynamic instability, as well as ischaemia-reperfusion injuries [36,37]. Total vascular exclusion can be performed with or without venous bypass [38]. The application of this technique in advanced iCCA cases has rarely been performed because of the complexity of the procedure and the dismal prognosis; however, it may prolong the OS in carefully selected cases [39].

## 5. Outcome

Hepatic resection with vascular reconstruction for the treatment of iCCA requires extensive experience in hepatobiliary surgical oncology and transplantation. This is confirmed by the fact that treatment at an academic centre has been associated with a lower incidence of a positive surgical margin, as well as decreased 90-day mortality and improved OS, among patients who undergo iCCA resection [40].

One previous clinical study reported that the immediate and long-term results were comparable in the groups with versus without PV and IVC resection [41]. This retrospective cohort study included 121 patients with iCCA who underwent major liver resection and examined their long-term outcomes. Among them, major vascular resection was performed in 14 (12%; IVC in 9, PV in 5). The authors reported that major postoperative complications (Dindo-Clavien ≥ 3) occurred in four (29%) patients in the vascular resection group versus 17 (16%) patients in the non-vascular group (*p* = 0.263). Postoperative death due to liver failure occurred in one patient in the vascular group. The median OS did not differ between patients treated with versus without vascular resection (32 vs. 49 months, respectively, *p* = 0.268). Despite the satisfactory comparative results of the study, the small number of observations leaves vascular resection a debatable issue.

Only two multi-centre studies have been published to date, both of which performed comparative analyses of the immediate and long-term results of patients with iCCA treated with versus without vascular resection.

In 2017, Reames et al. published a multi-institutional study of 1087 iCCA patients, including 128 (11.8%) who underwent major vascular resection. Despite more advanced disease, major vascular resection was not associated with the risk of any or major complications. This study showed comparable postoperative results, with reported mortality rates of 7% and 8% in patients undergoing hepatectomy with versus without concomitant major vascular resection, respectively. The 5-year OS rate was 36% in patients who required vascular resection versus 40% in those who did not. One limitation of this study was PV and IVC resection were pooled in the short- and long-term results [42].

Conci et al. [43] presented a multi-centre European study that included 270 patients with resected iCCA. The patients were divided into three groups: PV resection (PVR), IVC resection (IVCR), and no vascular resection (NVR). Thirty-one patients (11.5%) underwent VR: PVR in 15 (5.6%) and IVCR in 16 (5.9%). R0 resection rates were comparable among the three groups and achieved 73.%. The postoperative mortality rate increased in the VR group: 2.5% in the NVR group, 6.7% in the PVR group, and 12.5% in the IVCR group. The 5-year OS rates progressively decreased from 38.4% in the NVR group to 30.1% in the IVCR group and 22.2% in the PVR group (*p* = 0.030). However, the multivariable analysis did not confirm an association between VR and prognosis. The following prognostic factors were identified: size ≥50 mm, patterns of distribution of hepatic nodules (single, satellite, or multifocal), lymph node metastases (N1), and R1 resections. In the VR group, the 5-year OS rate in patients without lymph node metastases who underwent R0 resection (VRR0N0) was 44.4%, while that in N1 patients undergoing R1 resection was 20% (*p* < 0.001).

Both multi-centre studies showed comparative results in terms of vascular resection for iCCA (Table 1) [43].

Our experience at Miguel Servet University Hospital (Table 2) shows the 5-year cases of macrovascular invasion due to iCCA, for which the 1-year survival rate was only 40% and only one patient survived for 3 years.

## 6. Neoadjuvant and Adjuvant Therapy

As mentioned above, patients often have advanced disease at the time of diagnosis; therefore, in a high number of cases, an R1 resection cannot be avoided or the future remnant liver is insufficient. Moreover, in large or centrally located tumours, there is sometimes no more margin to be left, or, more often, the final histology shows microscopic tumour invasion of the resection margin that was intraoperatively assumed to be tumour-free, such as in tissues of the biliary, venous, parenchyma, or surrounding structures.

The effectiveness of current chemotherapy protocols and value of R1 resections may require re-evaluation, particularly in combination with neoadjuvant and downsizing therapy strategies, including intraoperative or intratumoural radiotherapy. Furthermore, in cases of unexpected R1 resection, the adjuvant therapy should be re-evaluated [44,45,46]. A randomized controlled multicentre study of capecitabine compared with observation in resected biliary tract cancer (BILCAP study) demonstrated perspectives of adjuvant therapy for iCCA. In this study, 447 patients were enrolled; 223 patients with biliary tract cancer resected with curative intent were randomly assigned to the capecitabine group, and 224 to the observation group. Although this study did not meet its primary endpoint of improving overall survival in the intention-to-treat population, the prespecified sensitivity and per-protocol analyses suggest capecitabine can improve overall survival in patients with resected biliary tract cancer when used as adjuvant chemotherapy following surgery and could be considered as standard of care [45]. Abou-Alfa et al., in 2020, conducted a multicentric phase 2 study with peginatimib in patients with previously treated, locally advanced, or metastatic cholangiocarcinoma with objective response [47].

Neoadjuvant therapy data are mainly limited to small size cohort studies with different treatment modalities—including radiotherapy, chemotherapy, chemoradiation, and local liver-directed therapies—and heterogeneous approaches, with a R0 resection rate ranging from 30 to 80% [48].

It is well known that radical resection alone features limited survival improvement. Although the results are clearly better than those of non-resected tumours, the achieved long-term survival is poor, even with recurrence, after extensive resection with complex reconstruction. This also coincides with the fact that centrally located tumours that require vascular reconstructions are usually large, which is associated with poor results [44].

Cases of iCCA with micro- and macrovascular involvement should be managed with multifocal treatment to improve the prognosis of this dismal disease [45,46].

## 7. Conclusions

Since resection offers the only chance for a cure, extended liver resection in combination with complex reconstruction of the vascular, biliary, or surrounding tissues seems justified for achieving complete tumour removal. However, despite aggressive surgery, tumour recurrence occurs frequently and the long-term oncological results are very poor.

Cases of iCCA with micro- and macrovascular involvement should be treated with multimodal therapy to improve their prognosis.

## Figures and Tables

**Table 1:** Multi-centre studies of vascular resections in iCCA.

Author	Year	Patients, *n*	Vascular Resection	Type of Vascular Resection, *n*	Postoperative Mortality, *n*	5-Year OS. Rate
Reams BN [42]	2017	1087	128 (11.8%)	PVR, 98 (9.8%) IVCR, 21 (1.9%); MVR, 9 (0.8%)	VR, 7%; PVR, n/a; IVCR, n/a	VR, 36%; PVR, n/a; IVCR, n/a
Conci S [43]	2020	270	31 (11%)	PVR, 15 (5.6%); ICVR, 16 (5.9%)	VR, 9.6%; PVR, 6.7%; CVR, 12.5%	PVR, 22.2%; ICVR, 30.1%

iCCA, intrahepatic cholangiocarcinoma; OS, overall survival; IVCR, inferior vena cava resection; MVR, multiple vascular resection; OS, overall survival; PVR, portal vein resection; VR, vascular resection.

**Table 2:** Miguel Servet University Hospital series of macrovascular involvement in cases of iCCA, 2016–2020.

	Sex	Tumoral Size (Maximum) mm	Multifocal	Radiological Vascular Involvement	Portal Vein (Histological)	IVC (Histol)	HV (Histol)	Nodes	Resection	TNM	1 Year-sv
68	Male	10.5	Yes	Yes	Yes	No	No	Negative	Left hepatectomy + PVR	pT2pN0M0	Yes
45	Male	3.7	Yes	Yes	Yes	Yes (resection)	No	Positive	Left hepatectomy + biliary duct + PVR	pT2pN1M0	No
73	Male	5.2	Yes	Yes	Yes	*	*	Positive	Left hepatectomy + PVR	pT2pN1M0	Yes
64	Male	6	No	Yes	No	No	No	Negative	Right trisectioriectomy + PVR + IVCR + LHVRC (bypass v.-v.)	pT4pN1M0	No
82	Female	6.2	No	Yes	Yes	*	*	Negative	S1-2-3-4-8 + PVR	pT2pN0M0	Yes
47	Male	6.5	No	Yes	Yes	*	*	Negative	Left hepatectomy + S1 + PVR	pT4pN2M0	Yes
73	Female	5.5	No	Yes	No	No	No	Positive	Left hepatectomy + IVCR + HV graft (bypass v.-v.)	ypT1bypN1M0	No
68	Male	12	No	Yes	Yes	No	No	Negative	Mesohepatectomy + colectomy + gastrectomy + PVR	pT4bpN1M1	No
69	Female	6	No	Yes	*	Yes (resection)	*	Negative	Left trisectoriectomy + IVCR (ALPPS)	pT2pN0M0	No
72	Female	5	No	Yes	Yes	*	*	Negative	Left hepatectomy + biliary duct + PVR	pT4pN1M0	No

iCCA, intrahepatic cholangiocarcinoma; bypass v.-v., veno-venous bypass; HV graft, iliac cadaveric artery from V6–V7 to the IVC; IVC, inferior vena cava; IVCR, inferior vena cava resection; LHVRC, left hepatic vein resection and reconstruction; PVR, portal vein resection; SV, survival; TNM, tumour node, Metastasis; ALPPS, associating liver partition with portal vein ligation for staged hepatectomy. * No histological sample (histol): positive vessel wall involvement.

## Data Availability

Not applicable.
